# Computational Characterization of Osteoporosis Associated SNPs and Genes Identified by Genome-Wide Association Studies

**DOI:** 10.1371/journal.pone.0150070

**Published:** 2016-03-01

**Authors:** Longjuan Qin, Yuyong Liu, Ya Wang, Guiju Wu, Jie Chen, Weiyuan Ye, Jiancai Yang, Qingyang Huang

**Affiliations:** 1 College of Life Sciences, Central China Normal University, Wuhan, 430079, China; 2 College of Computer Sciences, Central China Normal University, Wuhan, 430079, China; University of Vermont, UNITED STATES

## Abstract

**Objectives:**

Genome-wide association studies (GWASs) have revealed many SNPs and genes associated with osteoporosis. However, influence of these SNPs and genes on the predisposition to osteoporosis is not fully understood. We aimed to identify osteoporosis GWASs-associated SNPs potentially influencing the binding affinity of transcription factors and miRNAs, and reveal enrichment signaling pathway and “hub” genes of osteoporosis GWAS-associated genes.

**Methods:**

We conducted multiple computational analyses to explore function and mechanisms of osteoporosis GWAS-associated SNPs and genes, including SNP conservation analysis and functional annotation (influence of SNPs on transcription factors and miRNA binding), gene ontology analysis, pathway analysis and protein-protein interaction analysis.

**Results:**

Our results suggested that a number of SNPs potentially influence the binding affinity of transcription factors (NFATC2, MEF2C, SOX9, RUNX2, ESR2, FOXA1 and STAT3) and miRNAs. Osteoporosis GWASs-associated genes showed enrichment of Wnt signaling pathway, basal cell carcinoma and Hedgehog signaling pathway. Highly interconnected “hub” genes revealed by interaction network analysis are *RUNX2*, *SP7*, *TNFRSF11B*, *LRP5*, *DKK1*, *ESR1* and *SOST*.

**Conclusions:**

Our results provided the targets for further experimental assessment and further insight on osteoporosis pathophysiology.

## Introduction

Osteoporosis is a complex, polygenic disease that is characterized by reduced bone mass and microarchitecture deterioration of bone tissues, thereby leading to loss of strength and increased risk of fractures [1]. Osteoporosis and its main complication and fragility fractures incur substantial global morbidity and mortality [2]. The causes of osteoporosis and osteoporotic fractures are genetic background, environmental factors and their interactions [3]. Osteoporosis is defined clinically through the measurement of bone mineral density (BMD). BMD is highly heritable with the heritability estimates of 0.5–0.9 [3]. By contrast, heritability estimates of fractures are relatively low [3].

With the completion of the International HapMap Project [4] and 1000 Genomes Project [5], genome-wide association study (GWAS), a test for association between hundreds of thousands of single-nucleotide polymorphisms (SNPs) and a specific phenotype, becomes a major approach to discover genes and SNPs that contribute to complex diseases, and has been a resounding success [6]. By the end of 2014, nine GWAS [7–8] and nine meta-analyses [9–10] reported 107 genes and 129 SNPs (Lead SNP) that were associated with BMD, osteoporosis or fractures with a significance threshold of 5×10^−8^. Functional characterization and mechanistic elucidation of these SNPs and genes are next major challenge [11].

Tools of computational biology are powerful for post-GWAS studies, and could identify the potential and promising causal SNPs that deserve experimental test for follow-up functional studies. Extensive work has been done in this area in recent years [12,13]. Various computational methods and tools have been successfully developed and are available through the World Wide Web. Their performances were well validated through identifying numerous disease-associated SNPs for further study and revealing previously unknown mechanisms for complex diseases. For example, Zhang et al. [14] reported an immune- and microglia-specific module that is dominated by genes involved in pathogen phagocytosis, contains TYROBP as a key regulator, and is upregulated in late-onset Alzheimer’s disease through an integrative network-based approach. Integrative computational analysis of cross-species conservation with an assessment of co-occurring transcription factor (TF) binding sites suggested that the T allele of rs4684847 can reduce the binding ability of the transcriptional regulator PRRX1 and maintains a higher level of *PPARG2* expression, while the risk C allele enhances binding of the PRRX1, and thus inhibits *PPARG2* mRNA expression, thereby provoking dysregulation of free fatty acids turnover and glucose homeostasis [15]. Computational analyses currently serve as an useful starting point to guide the design of functional assays [12].

Recent studies demonstrated that SNPs could contribute to osteoporosis susceptibility by influencing TF or miRNA binding. The rs4317882 of *MPP7* is associated with BMD with the genome-wide significance. EMSA results showed the binding of TF GATA2 to the risk allele 'A' but not the 'G' allele of rs4317882 [16]. Several SNPs of *POSTN* were associated with BMD or vertebral fractures. A specific binding of CDX1 to the sequence with the major allele A but not the variant G allele of the *POSTN* rs9547970 was confirmed also by EMSA [17]. Three SNPs (rs6854081, rs1048201 and rs7683093) in miRNA target sites of the *FGF2* gene were significantly associated with femoral neck BMD [18]. The rs17737058 significantly associated with the decreased BMD is disruptive to the miR-488-5p:NCOA1 interaction [19]. In this study, we applied multiple computational approaches to comprehensively and systematically analyze osteoporosis-associated SNPs and genes identified by GWAS, including gene ontology (GO) and pathway analysis, protein-protein interaction analysis, SNP conservation analysis and functional annotation (effects of SNPs on TF binding and miRNA binding). We aimed to identify potential SNPs for follow-up functional analyses, and to offer guidance for future research with regard to the etiology and pathogenesis of osteoporosis.

## Materials and Methods

### Search strategy and data collection

**Fig 1:**
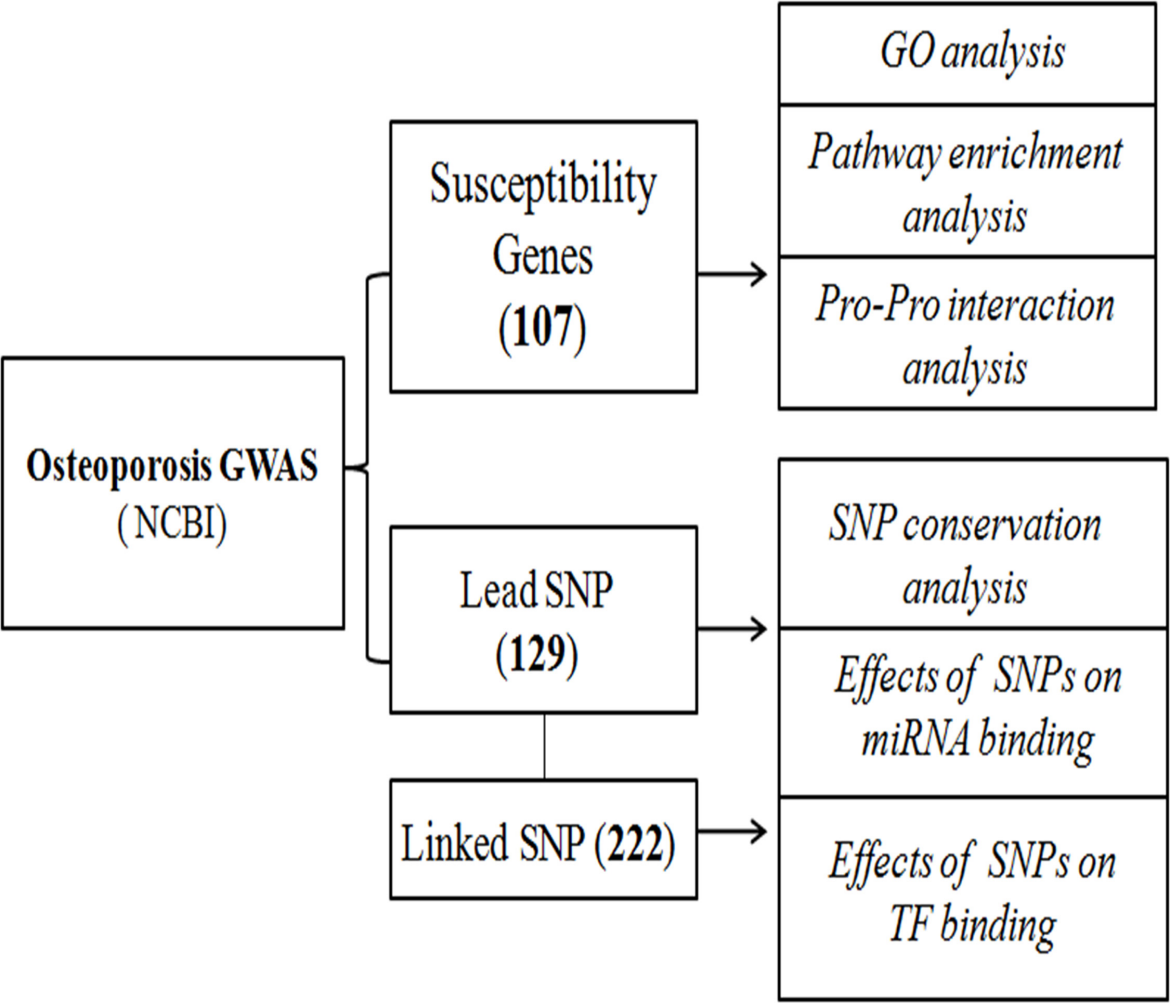
The workflow of osteoporosis-associated loci identified by GWAS.

We searched osteoporosis GWAS articles using the following keywords: “Osteoporosis”/“BMD”/“fracture” and “GWAS”, and extracted osteoporosis GWAS associated SNPs with P<5×10^−8^ and genes (updated to May 1, 2015). We also searched the linked SNPs which were in linkage disequilibrium (LD) (D’>0.8) with osteoporosis GWAS associated lead SNPs using LD information in the Caucasians population via HapMap website. A detailed workflow chart of our methodology is illustrated in Fig 1.

### Conservation analysis

Sequence conservation is a signal of functional constraint, and can be used for function prediction. Comparative genomics is an evolutionary approach to detect functional elements [20]. The VISTA (http://pipeline.lbl.gov/cgi-bin/gateway2) and UCSC Genome Browser (http://genome.ucsc.edu) are two web-based tools that can be used to align multiple sequences from several vertebrate species, including humans (hg19: GRCh37). The position of SNP was submitted to the VISTA detabase [21], and Chimp (panTro4: CSAC 2.1.4) and Mouse (mm10: GRCm38) genomes were selected. The p-values were obtained using the rankVISTA (http://genome.lbl.gov/vista/rankVISTA.shtml) tool. The UCSC Genome Browser [22] was used to compute conservative frequency for individual base of SNPs. Table 1 showed the number of species with the conserved major human allele out of nine default organisms (human, rhesus, mouse, dog, elephant, chicken, x. tropicalis, zebrafish and lamprey) from the vertebrate Multiz alignment of 100 species [23].

**Table 1:** Conservation analysis of osteoporosis GWAS-associated lead SNPs.

SNPs	VISTA		UCSC
	Mouse (*P*)	Chimp (*P*)	
rs7741021	1.10E-03	-	6/9
rs4355801	4.60E-03	0.1	5/9
rs7071206	-	9.10E-08	5/9
rs227425	8.20E-05	-	5/9
rs9287237	-	1.80E-10	4/9
rs2952559	-	6.90E-03	4/9
rs3801387	7.30E-20	4.60E-03	4/9
rs4233949	1.80E-03	-	4/9
rs9533095	-	1.40E-02	4/9

### Influence of SNPs on TF binding

Interactions between TFs and their DNA binding sites (TFBS) play a key role in gene transcription. SNPs that may locate within the promoter or distant enhancer region of gene can alter (modify, destroy or create) the binding of TFs with DNA, and subsequently regulate gene expression. The JASPAR database (http://jaspar.cgb.ki.se) [24] was utilized to predict the candidate SNPs which may change the binding affinity. The JASPAR CORE database contains a curated and non-redundant set of profiles, derived from published collections of experimentally defined TFBSs for eukaryotes. The 51 bp DNA sequence surrounding the SNP was submitted to the JASPAR CORE (2014) database to analyze the functionally potential SNPs that may disrupt the binding of TFs or change the binding affinity (only human matrix models were selected). The score of a sequence suggest its potential as putative TFBS. A string threshold of 5:0 was used to define loss or gain for a SNP to filter false positive results.

### Effects of SNPs on miRNA binding

miRNAs regulate gene expression by binding target mRNAs. miRNAs predominantly decrease mRNA stability through base pairing with the 3’UTR of target mRNAs. The recognition of target mRNA by miRNA involves a small complementary sequence from 2 to 7 nucleotides long. Recognition sequence alteration by SNPs can either generate or destroy miRNA binding sites in mRNAs. MiRNA related SNP database (miRNASNP v2.0, http://www.bioguo.org/miRNASNP2/online.php) provides a resource of the miRNA-related SNPs, which included SNPs in human pre-miRNAs and miRNA flanks, SNPs in other species' miRNAs, and target gain or loss by SNPs in miRNA seed regions or 3'UTR of target mRNAs. We used miRNASNP to predict the influence of SNPs on the miRNA binding.

### GO and pathway analysis

Gene Ontology (GO, http://www.geneontology.org) is a widely adopted source of gene functional annotation including biological process, molecular function and cellular component. Kyoto Encyclopedia of Genes and Genomes (KEGG, http://www.genome.jp/kegg/pathway.html) is a database resource for understanding high-level functions and utilities of the biological system. We used Search Tool for the Retrieval of Interacting Genes (STRING, version 9.1, http://string-db.org/) to investigate if any common functional trends are associated with the osteoporosis GWAS-associated genes (P<5x10^-8^). The hypergeometric statistical test was used to determine if a pathway from GO or KEGG is significantly enriched. P value for each pathway was adjusted with Benjamini & Hochberg method to correct for multiple testing [25]. GO and KEGG pathways with an adjusted *P*<0.01 were considered to be significant [26]. In addition, PANTHER (Protein Analysis Through Evolutionary Relationships) classification system, which can provide intuitive visualizations of images of GO analysis, was used to classify proteins (and their genes) in order to facilitate high-throughput analysis.

### Protein-protein interaction analysis

STRING is a database of known and predicted protein interactions, including direct (physical) and indirect (functional) associations. STRING quantitatively integrates interaction data derived from four sources: Genomic Context, High-Throughput Experiments, Co-expression (Conserved) and Previous Knowledge [27]. The database currently covers 5,214,234 proteins from 1133 organisms. STRING was used to generate risk gene (protein) network.

### Functional annotation of SNPs using GWAS3D

Interpreting disease associated non-coding SNPs is an indispensable step to understand molecular mechanism of complex diseases. GWAS3D (http://jjwanglab.org/gwas3d) systematically computes the probability of GWAS associated SNPs affecting regulatory pathways and underlying disease associations by integrating chromatin state, functional genomics, sequence motif and conservation information [13]. It also provides comprehensive annotations and visualizations to help users interpret the results. In this study, we used GWAS3D to identify the most significant SNPs which has a long-range interaction signal with other locus with the following parameters: r^2^ = 0.8, p-value cutoff 1×10^−5^, HapMap I+II+III, CEU population, binding site p-value 0.01, promoter range from 100 to 500bp, 30 variant size and 3 interaction size.

## Results

### Osteoporosis GWAS-associated gene/SNPs

According to NCBI association results, a total of 129 SNPs and 107 genes were associated with BMD, osteoporosis or fractures with a significance threshold of 5×10^−8^. Of these SNPs, 72 mapped to introns, 35 to intergenic regions, 6 to exons, and 3 in 3’ UTR. We identified 222 SNPs linked with osteoporosis GWAS-associated lead SNPs using LD information in the Caucasians population via HapMap website. The detail of osteoporosis GWAS-associated lead SNPs and genes was shown in S1 Table.

### Conservation analysis of osteoporosis-associated SNPs

To investigate the evolutionary conservation of osteoporosis GWAS-associated SNPs, we performed a comparative genome analysis using VISTA. Fourteen SNPs are within conserved sequence in the pairwise alignments of Mouse (*P*<0.05), whereas the number of conservative SNPs increases to 20 with Chimp. We estimated the evolutionary conservation rate of SNPs using UCSC Genome Brower. Twenty-six SNPs might locate in evolutionally conservative regions with the conservative frequency of single base >4/9. Nine SNPs are significantly conservative in both methods (Table 1).

### Influence of SNPs on TF binding

Effects of osteoporosis GWAS-associated lead SNPs and their linked SNPs on the TF binding affinity were analyzed by JASPAR. Top potentially affected TFs were SRY, SPIB, JUN::FOS, THAP1 and NFATC2. Table 2 showed the results (scores >5) of TFs NFATC2 (31), MEF2C (21), SOX9 (20), RUNX2 (11), ESR2 (9), FOXA1 (5) and STAT3 (4), which are implicated in bone metabolism, bone-related cell development and differentiation. RUNX2 and MEF2C are two important TFs which involved in the regulation of bone development and metabolism, whereas STAT is an important member component of Jak-STAT signaling pathway. FOXA1 and ESR are required for estrogen action in the osteoporosis.

**Table 2:** Effects of SNPs on the binding of TF [gain (+) or loss (-)].

TFs	Number		Lead SNPs	Linked SNPs
NFATC2	+	16	rs344081,rs271170,rs4869739 rs4729260,rs16921914,rs9533095 rs9303521	rs1741,rs1135999,rs11650567,rs17408318 rs17457484,rs4869739,rs638076,rs6904261 rs6962748
	-	15	rs1430742,rs2566755,rs1463104,rs2887571	rs2124954,rs17154676,rs9309078 rs13345828,rs227421,rs11627052 rs11757289,rs399996,rs4335155,rs7221132 rs9466057
MEF2C	+	10	rs1463104,rs479336,rs9287237 rs6894139,rs1286083	rs12474135,rs2124954,rs271171,rs2812415 rs6984675
	-	11	rs17509082,rs87938,rs10048146 rs6930633	rs227427,rs4487685,rs4922787,rs1286079 rs9998083,rs1989565,rs158143
SOX9	+	10	rs2707466,rs718766,rs1286083 rs2273061,rs1366594	rs9383935,rs17013324,rs4922787,rs2707466 rs2536175
	-	10	rs13247600,rs479336	rs9371552,rs11627052,rs1828720,rs7683315 rs11844519,rs271171,rs6932260,rs6744982
RUNX2	+	7	rs7084921,rs3801387	rs1471400,rs985296,rs11773399,rs11844519 rs11155797
	-	4	rs7932354	rs9466058,rs2207491,rs6984675
ESR2	+	3	-	rs9646629,rs11658881,rs219749
	-	6	rs1430740,rs6993813,rs7117858 rs2887571	rs13177576,rs2490058
FOXA1	+	1	rs1053051	-
	-	4	rs4729260	rs9998083,rs7155014,rs7792993
STAT3	+	1	-	rs11752730
	-	3	-	rs6465511,rs228768,rs3760891

### Influence of osteoporosis GWAS-associated SNPs on miRNA binding

Using miRNASNP v2.0, we have predicted 2 GWAS-revealed lead SNPs (rs884205 and rs1026364) that may influence the recognition and targeting of miRNA. The rs884205 C and rs1026364 T alleles create the binding site for miR-3658 and miR-345-5p, respectively. The number of candidate SNPs increased to 11 when predicted linked SNP (222) (Table 3).

**Table 3:** Effect of SNPs on the binding of miRNA (gain or loss).

SNP/Gene	miRNA (loss)	miRNA (gain)
*Lead SNP*		
rs884205(A→C)/ *TNFRSF11A*		miR-3658
rs1026364(G→T)/ *KIAA2018*		miR-345-5p
*Linked SNP*		
rs3204848(A→G)/ *C12orf23*	miR-493-5p/miR-4718	
rs3734805(A→C)/ *CCDC170*	miR-641/3617-5p/miR-3915	miR-4524b-3p/miR-4459
rs6932260(T→C)/ *CCDC170*	miR-16-2-3p/miR-195-3p/miR-656	
rs6904261(G→A)/ *CCDC170*		miR-4778-3p
rs6932603(T→C)/ *CCDC170*	miR-153/miR-544a	
rs9383935(C→T)/ *CCDC170*	miR-4798-3p	miR-5197-5p
rs3734806(G→A)/ *CCDC170*		miR-2964a-5p/miR-572
rs3757322(T→G)/ *CCDC170*		miR-4274/miR-374b-3p
rs1741(G→C)/ *PDXDC1*		miR -548t-5p/miR-4536-5p/miR-4796-3p/miR-548az-5p
rs6498540(A→G)*/ PDXDC1*	miR-3156-3p	miR-1260a/miR-1260b
rs1121(G→A)/ *PDXDC1*		miR-550a-5p/miR-550a-3-5p/miR-1271-3p/miR-3064-5p/miR-6504-5p

### GO and pathways analyses of osteoporosis GWAS-associated genes

**Fig 2:**
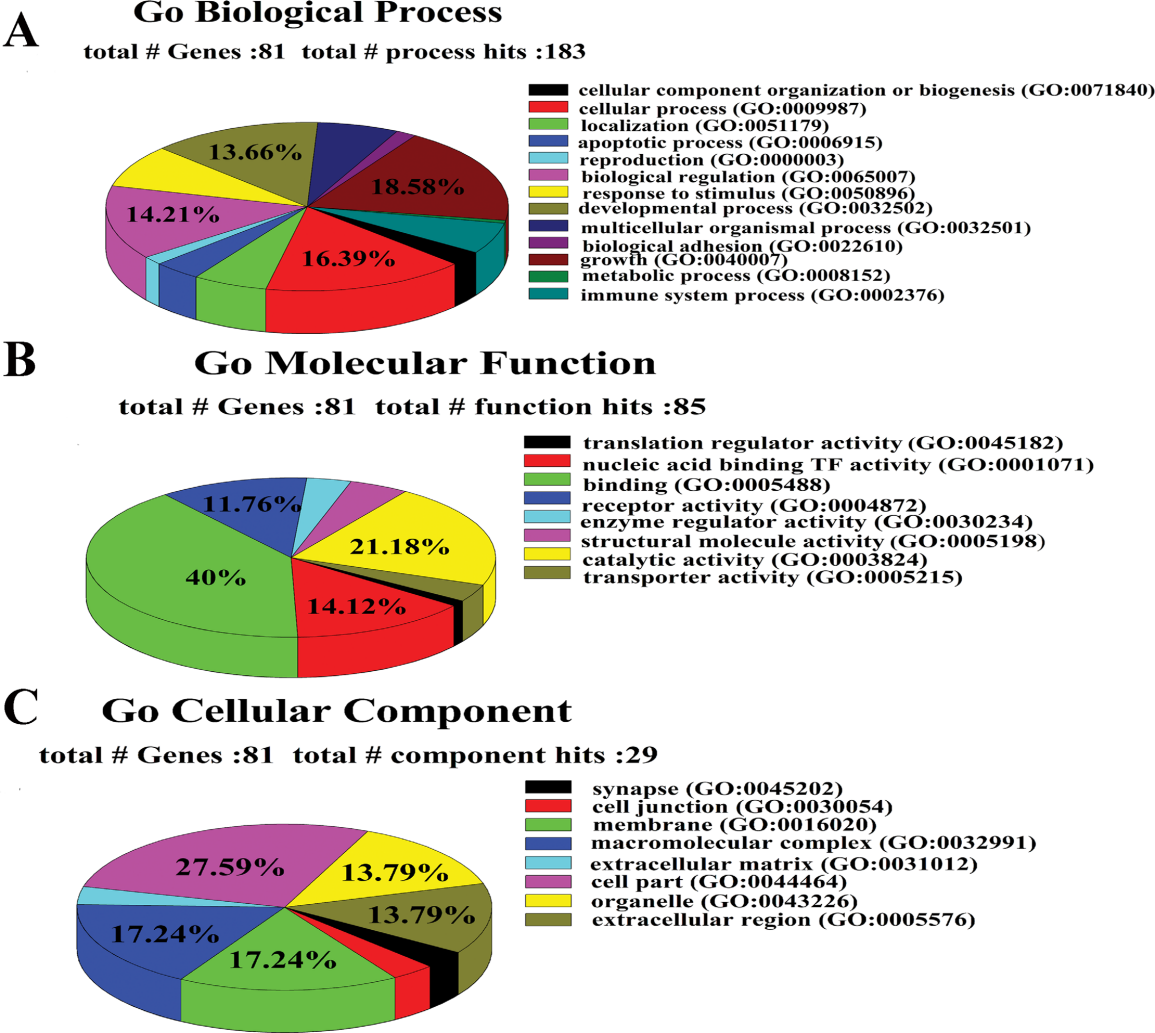
**GO annotation of osteoporosis GWAS-associated gene by PANTHER** (A: Biological Process; B: Molecular Function; C: Cellular Component).

The results of osteoporosis GWAS-associated genes enrichment by PANTHER was shown in Fig 2. Distribution of genes conferring biological processes (Fig 2A) was as the following, metabolic process (34 genes, 18.6%), cellular process (30 genes, 16.4%), biological regulation (26 genes, 14.2%), developmental process (25 genes, 13.7%), response to stimulus (15 genes, 8.2%), multicellular organismal process (12 genes, 6.6%), localization (11 genes, 6%), immune system process (11 genes, 6%), apoptotic process (7 genes, 3.8%), cellular component organization or biogenesis (5 genes, 2.7%), reproduction (3 genes, 1.6%), biological adhesion (3 genes, 1.6%) and growth (1 genes, 0.5%). Genes for molecular functions (Fig 2B) were for binding (34 genes, 40.0%), catalytic activity (18 genes, 21.2%), receptor activity (10 genes, 11.8%), nucleic acid binding transcription factor activity (12 genes, 14.1%), structural molecule activity (4 genes, 4.7%), enzyme regulator activity (3 genes, 3.5%), transporter activity (3 genes, 3.5%) and translation regulator activity (1 genes, 1.2%). Genes for cellular components involved in cell part (8 genes, 27.6%), macromolecular complex (5 genes, 17.2%), membrane (5 genes, 17.2%), extracellular region (4 genes, 13.8%), organelle (4 genes, 13.8%), extracellular matrix (1 genes, 3.4%), cell junction (1 genes, 3.4%) and synapse (1 genes, 3.4%) (Fig 2C).

Pathway enrichment analysis of osteoporoses GWAS-associated genes in KEGG using STRING uncovered some evidence of over-representation of Wnt signaling pathway, Basal cell carcinoma and Hedgehog signaling pathway with adjusted *P*<0.05 (Table 4). It is well-established that Wnt and Hedgehog signaling pathway plays a key role in bone metabolism and osteoporosis pathogenesis.

**Table 4:** Pathway enrichment analysis of osteoporosis GWAS-associated genes.

GO_ID	Term	Number of genes	p-value
hsa04310	Wnt signaling pathway	6	2.48E-03
hsa05217	Basal cell carcinoma	4	8.49E-03
hsa04340	Hedgehog signaling pathway	4	9.12E-03

### Protein-protein interaction network

**Fig 3:**
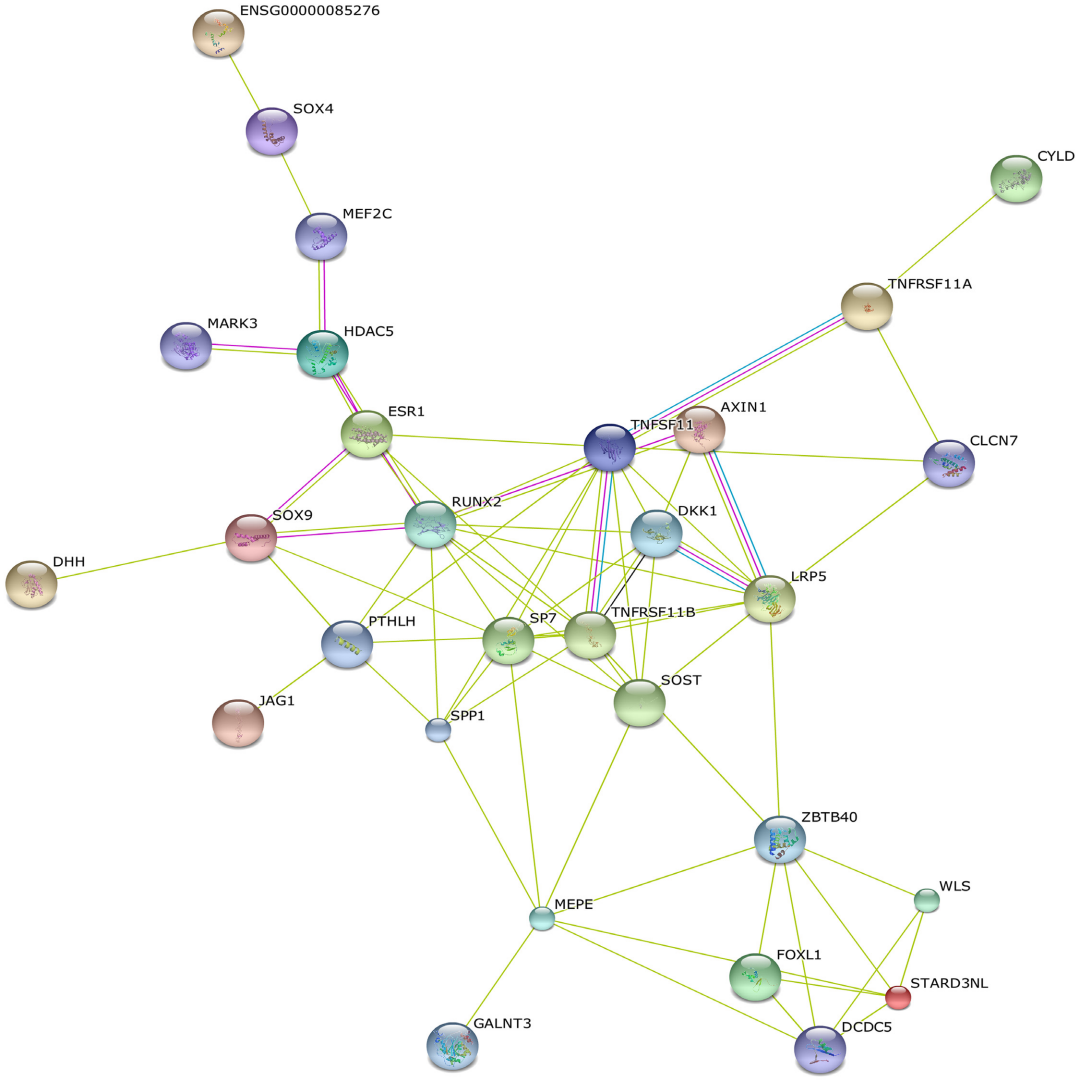
Protein-protein interaction network of osteoporosis GWAS-associated genes. The nodes and edges represent the proteins (genes) and interactions, respectively. Green means neighborhood, red means gene fusion, mustard means textmining, black means coexpression, purple means experiments, and blue means cooccurence.

Protein-protein interaction network analysis of 107 osteoporosis GWAS-associated genes showed significant connectivity among proteins using STRING (9.1) with default settings (observed interaction, 70; expected interaction, 8.83; *P*<1.00E-10; proteins, 76). Fig 3 shows the protein-protein interaction network by "evidence view" from STRING. Hub proteins with the strong connections were RUNX2 (12), TNFRSF11B (10), LRP5 (9), SP7 (10), SOST (7), DKK1 (7), ESR1 (5). These proteins were involved in the Wnt signaling (LRP5, SOST, DKK1), RANK-RANKL-OPG pathway (TNFRSF11B), mesenchymal stem cell differentiation (RUNX2, SP7) and hormone signaling (ESR1).

### Long-range interaction of osteoporosis-associated SNPs using GWAS3D

**Fig 4:**
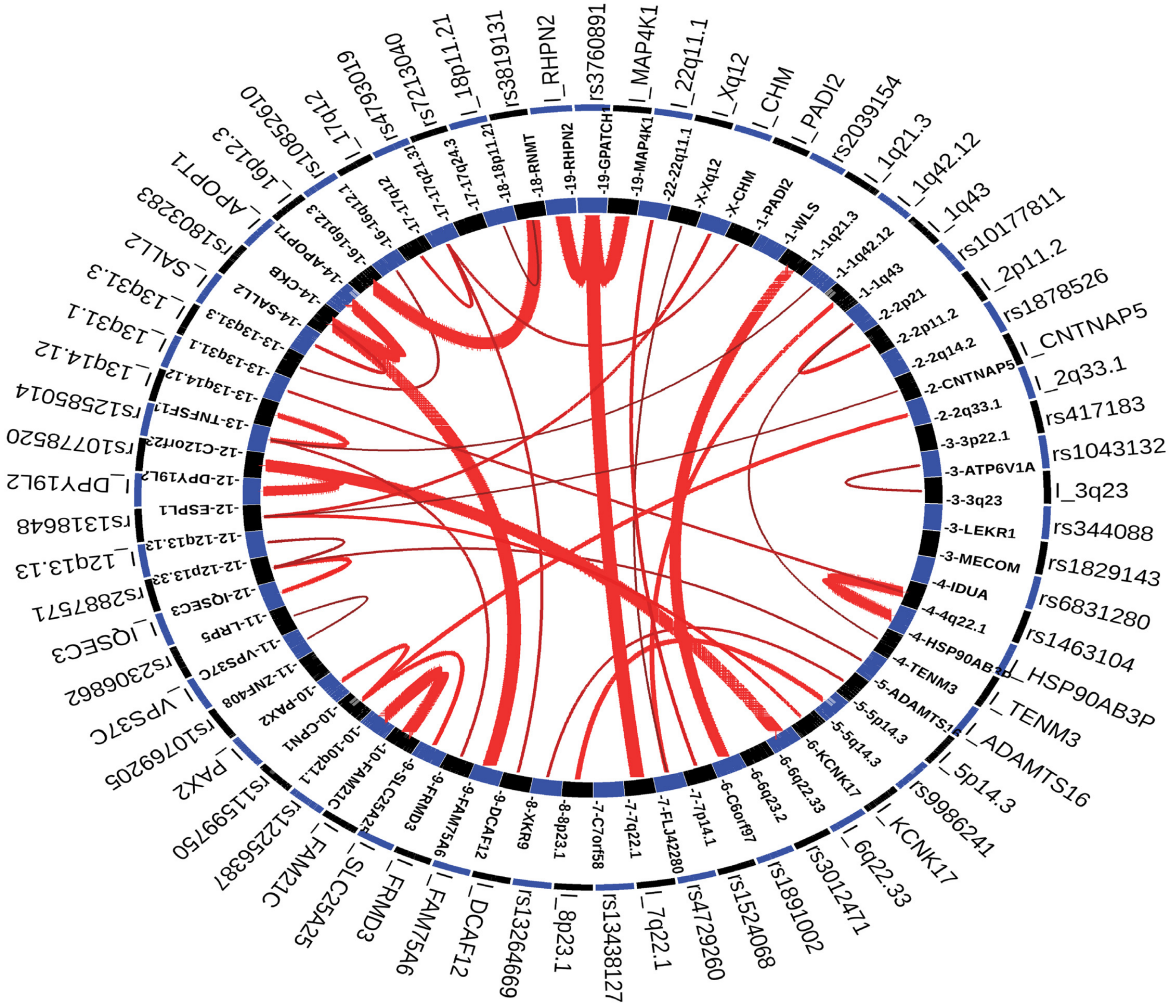
The circle plot of GWAS3D for osteoporosis GWAS-SNPs based on all cell line and CEU population. The red line indicated long-range interaction signals, and the intensity of interaction was represented by the width of the line. Interactive elements with significant SNP will start with ‘I_’.

SNPs rs1463104, rs4729260 and rs2887571 were detected as significant SNPs by GWAS3D based on the CEU population and all cell types listed in GWAS3D (Fig 4). SNP rs1463104, located on the downstream of *MEPE* intronic region of *DECR1* in chromosome 4, has two long-range interaction signal with locus 13q31.1 and locus near *HSP90AB3P*. SNP rs4729260, located on the intronic region of *C7orf76* in chromosome 7, has two long-range interaction signals with locus Xq12 and 1q43. SNP rs2887571, located on the intergenic region of *ERC1* and *WNT5B* in chromosome 12p13.33, showed three long-range interaction signals with locus 12p13.13, locus near *IQSEC3* and *MON2*, respectively.

## Discussion

To explore function and mechanisms of osteoporosis GWAS-associated SNPs and genes, we characterized SNPs conservation and their influence on TFs and miRNA binding, and conducted GO and pathway analyses, protein-protein interaction analysis of osteoporosis GWAS-associated genes. Our analyses revealed nine significantly conservative SNPs. However, a number of SNPs potentially altering the binding affinity of TFs and miRNAs were not located within conservative regions, mirroring the controversy about relationship between conservation and functionality [17].

Our results indicated that SNPs may change the binding affinity of TFs NFATC2, MEF2C, SOX9, RUNX2, ESR2, FOXA1 and STAT3. RUNX2 and MEF2C are important TFs in bone metabolism. Multiple genes regulated by RUNX2 and MEF2C are involved in osteoblast differentiation and bone metabolism. MEF2C can bind to the ECR5 enhancer upstream of *SOST*, and regulate *SOST* expression [28]. It is well-established that Nfat plays a role in osteoclastogenesis. NFAT and Osterix cooperatively control osteoblastic bone formation [29]. Overexpression of a constitutively active form of *Nfatc2* suppresses osteoblastic gene expression *in vitro* [30]. *Nfatc2* activation in osteoblasts inhibits bone formation and causes cancellous bone osteopenia [31]. SOX9 is a master regulator TF in cartilage, and is essential for cartilage development in mice [32]. STAT is an important member component of Jak-STAT signaling pathway which plays an important role in bone development and metabolism. Among all the STATs, STAT3 is probably the most important TF mediating intracellular signaling in osteoblasts and osteoclasts. Selective inactivation of STAT3 in osteoblasts causes smaller body mass and lower bone mass due to inhibition of bone formation [33]. Clinical studies indicate that STAT3 mutation increases osteoclast number and bone resorption [34, 35]. Estrogen is a key hormonal regulator of bone metabolism [36]. The biological actions of estrogen are primarily mediated through estrogen receptor. Recent chromatin immunoprecipitation coupled with microarray studies postulate that the binding of ESR at nearly 50% of target sites on chromosomes is modulated by FOXA1 [37]. Interestingly, we found that SNPs influence both FOXA1 and ESR binding in multiple genes, providing a mechanistic link between risk SNPs and osteoporosis.

There are several successful examples of identification of the SNPs that alter miRNA binding and drive complex diseases [11]. Studies have indicated that miRNAs play a critical role in bone formation [38]. Our results indicated that several SNPs potentially influenced miRNA binding to target mRNA, providing important clues and targets for further experimental test.

Notably, our pathway enrichment analyses of osteoporosis GWAS-associated genes only confirmed well-known Wnt and Hedgehog signaling pathways, and failed to provide support for many other mechanisms previously promoted on the basis of physiological evidence as likely contributors to osteoporosis pathogenesis, presumably due to poor representation of the processes critical to osteoporosis development in existing pathway databases. Using STRING, we identified highly-interconnected network “hub genes (proteins)”, *RUNX2*, *SP7*, *TNFRSF11B*, *LRP5*, *ESR1*, *DKK1*, and *SOST*. LRP5, SOST and DKK1 are important members in Wnt signaling pathway. SP7 (Osterix) and RUNX2 are two specific TFs of osteoblast, and the central regulatory factors in osteoblast differentiation and function. RUNX2 binds to the promoters of a variety of genes (including osteocalcin, osteopontin, bone sialoprotein and type I collagen) related to osteoblast differentiation or function [39]. The expression of many of the bone related genes still require SP7, regardless of the presence of RUNX2 [40]. TNFRSF11B (OPG) is a member of the TNF-receptor superfamily and an osteoblast-secreted decoy receptor that functions as a negative regulator of bone resorption. OPG expression promotes osteoclast development and enhances its function under the condition of estrogen deficiency [41]. ESR1 are hormone regulators of bone metabolism. More importantly, network analyses also highlighted notable biological connections between osteoporosis GWAS-associated gene sets. For example, *SOST* is a negative regulator of bone formation and regulated by PTH and estrogen. Our network analyses showed that *SOST* is a direct target of RUNX2 and SP7.

In conclusion, computational characterization of osteoporosis GWAS-associated genes highlighted genes (proteins) and pathways that are crucial for osteoporosis pathophysiology, thereby improving our understanding of the condition and providing potential treatment targets. Our results also unraveled the potential functional mechanisms of osteoporosis GWAS-associated SNPs through influencing TF and miRNA binding that warrant further experimental test in the future.

## Supporting Information

S1 Table Information of osteoporosis GWAS-associated SNPs and genes. (XLS)
